# Achievement of specified lipid and high-sensitivity C-reactive protein levels with two statins in Chinese patients with hypercholesterolaemia

**DOI:** 10.1186/s12944-015-0116-0

**Published:** 2015-09-13

**Authors:** Cheng Ding, Miao Hu, Yong-Jian Wu, Brian Tomlinson

**Affiliations:** State Key Laboratory of Cardiovascular Disease, Department of Cardiology, Cardiovascular Institute, Fuwai Hospital and National Center for Cardiovascular Diseases, Chinese Academy of Medical Sciences & Peking Union Medical College, Beijing, China; Department of Medicine and Therapeutics, The Chinese University of Hong Kong, Prince of Wales Hospital, Shatin, Hong Kong

**Keywords:** High-sensitivity C-reactive protein, Statins, Low-density lipoprotein cholesterol, Chinese

## Abstract

**Background:**

Statins reduce plasma low-density lipoprotein cholesterol (LDL-C) and high-sensitivity C-reactive protein (hsCRP) levels. Rosuvastatin 10 mg daily appears to be more potent in reducing LDL-C than simvastatin 40 mg, but the relative effect of these two statin doses on hsCRP is unknown.

**Methods:**

Chinese hyperlipidaemic patients with high cardiovascular risk or familial hypercholesterolaemia (FH) were treated with rosuvastatin 10 mg and simvastatin 40 mg daily in an open-label crossover study. Lipid profiles were measured off treatment and after at least 4 weeks treatment with each of the two statins and hsCRP levels were measured on treatment with both statins.

**Results:**

Both treatments were well tolerated in 247 patients (age 55.7 ± 11.1 years; 100 male; 140 with FH) with good treatment compliance. There were statistically significant differences (*P* < 0.001) for rosuvastatin versus simvastatin for LDL-C reduction (−52.4 ± 11.9 % vs. -47.7 ± 10.8 %) and on-treatment LDL-C (2.62 ± 0.99 mmol/L vs. 2.86 ± 0.97 mmol/L), respectively, but the on-treatment hsCRP levels (1.33 ± 1.37 mg/L vs. 1.41 ± 1.57 mg/L, *P* > 0.05) were not significantly different. The lipid target (LDL-C <2.6 mmol/L) was achieved by 52.9 % with rosuvastatin compared with 42.6 % with simvastatin (*P* < 0.05). The proportions of patients attaining hsCRP targets of <2 and <1 mg/L were similar with the two statins (57.1 % and 74.6 % for rosuvastatin vs. 57.1 % and 80.1 % for simvastatin, *P* > 0.05).

**Conclusion:**

A significantly greater proportion of patients achieved LDL-C targets with rosuvastatin 10 mg compared to simvastatin 40 mg in Chinese patients with hypercholesterolaemia, but there was no significant difference in achieving hsCRP target levels with the two statins.

## Introduction

The significant cardiovascular benefits of statins have been proven in many large randomized clinical trials and these benefits are thought to be largely mediated by their effect on reducing plasma low-density lipoprotein cholesterol (LDL-C) concentrations. Other pleiotropic effects such as anti-inflammatory and anti-oxidative effects may also be involved [1, 2]. These large clinical trials showed that every 1 mmol/L reduction in LDL-C with statin therapy was associated with a proportional reduction of about 20 % in major vascular events [3] and more intensive statin therapy with greater reductions in LDL-C of 2–3 mmol/L might reduce the incidence of major vascular events by 40-50 % [4]. Based on this accumulated evidence, lipid guidelines have advocated lower LDL-C targets [5–7].

Inflammation plays a fundamental role in the pathogenesis of atherothrombotic disease [8, 9]. Prospective observational studies showed that a raised concentration of high-sensitivity C-reactive protein (hsCRP), a well-known and extensively studied systemic biomarker of inflammation, was associated with increased risk of vascular disease and mortality [10], although whether this association is causal or indicative of another underlying risk factor is still controversial [11]. Statin therapy reduces hsCRP levels in a wide range of patients and some studies demonstrated that patients who had lower hsCPR levels with statin therapy had greater clinical benefits [12–14]. The large JUPITER (Justification for the Use of statins in Primary prevention: an Intervention Trial Evaluating Rosuvastatin) study has shown that the reductions in LDL-C and hsCRP with rosuvastatin treatment were independent predictors of the vascular benefits of statin treatment and the reductions in LDL-C and hsCRP in individual patients were not significantly correlated suggesting that the change in hsCRP levels with statin is independent of the change in LDL-C [14]. The result of the JUPITER trial prompted the United States Food and Drug Administration to approve the use of rosuvastatin in subjects (>50 years in men; >60 years in women) with elevated hsCRP levels (>2 mg/L) and at least one additional cardiovascular risk factor in early 2010 [15].

Simvastatin and rosuvastatin are two commonly used statins worldwide with the doses of 40 mg simvastatin or 10 mg rosuvastatin being commonly prescribed in Chinese patients with increased cardiovascular risk and higher levels of LDL-C. Lower doses of 2.5 and 5 mg rosuvastatin are considered more standard in Japan and have been shown to slow coronary plaque progression [16], but higher doses are probably needed to produce regression of coronary plaque volume [17]. It has been shown that on average rosuvastatin 10 mg daily has a more potent effect on reducing LDL-C than that of simvastatin 40 mg [18, 19], but there is no study comparing the effect of these two statins on hsCRP which may involve pathways which differ from those involved in lowering LDL-C. This study examined the LDL-C and hsCRP goal attainment with simvastatin and rosuvastatin in Chinese patients with hypercholesterolaemia.

## Methods

This study analysed data from Chinese patients with hypercholesterolaemia who received both rosuvastatin and simvastatin in an open-label, crossover study performed to assess the pharmacogenetics of responses as described previously [18]. In brief, the study subjects were Hong Kong Han Chinese patients aged at least 18 years with established coronary heart disease (CHD) or CHD risk equivalent [6], including some with heterozygous familial hypercholesterolaemia (FH). Individuals with uncontrolled diabetes, hypertension or thyroid disease, significant renal or hepatic dysfunction, those who had experienced a cardiovascular disease (CVD) event within the 3 months before recruitment, or those with poor adherence with statin therapy (reporting taking <80 % of the prescribed number of tablets) were excluded. There were 177 patients taking rosuvastatin 10 mg daily and 70 patients taking simvastatin 40 mg as initial treatment. Baseline and on-treatment lipid profiles and on-treatment hsCRP levels were measured after at least 4 weeks treatment for each statin. Thereafter, patients were switched to the other statin for at least 4 weeks with lipid and hsCRP levels being re-measured.

The study protocol was approved by the Clinical Research Ethics Committee and the study was performed in accordance with the Declaration of Helsinki. All participants gave their written informed consent.

### Laboratory analysis

The lipid and laboratory safety parameters were measured by routine methods. The plasma hsCRP concentration was determined using an immunonephelometric method (Siemens Dade Behring CardioPhase hsCRP assay, Newark, DE, USA) on the Siemens BN ProSpec® System with the detection limit of 0.146 mg/L (the measurement range was 0.146–9.35 mg/L) and the inter-assay coefficients of variation of 2.5, 3.8 and 2.1 % at hsCRP concentrations of 0.500, 1.30 and 2.10 mg/L, respectively.

### Statistical analysis

Subjects with hsCRP levels >10 mg/L were excluded from the analysis for hsCRP (*n* = 13) as this high level of hsCRP is likely to be due to acute illness and for those with hsCRP levels below the limit of detection, the value of 0.1 mg/L was assigned. The on-treatment LDL-C and hsCRP levels with the two statins and the percentage change in lipids were compared by paired *t*-test or Wilcoxon test wherever appropriate. According to the Adult Treatment Panel III guideline, the overall LDL-C goal for high-risk patients is <2.6 mmol/L with an optional intensified LDL-C goal of <1.8 mmol/L for patients with very high-risk [7]. Subjects were evaluated as to whether they had attained the hsCRP goals of <2 mg/L or <1 mg/L. The LDL-C and hsCRP goal attainments with the two statins were compared by using a *χ*^2^ test. *P* value <0.05 was considered statistically significant. Data were analyzed with SPSS version 17.0 (SPSS Inc., Chicago, IL, USA).

## Results

**Fig. 1:**
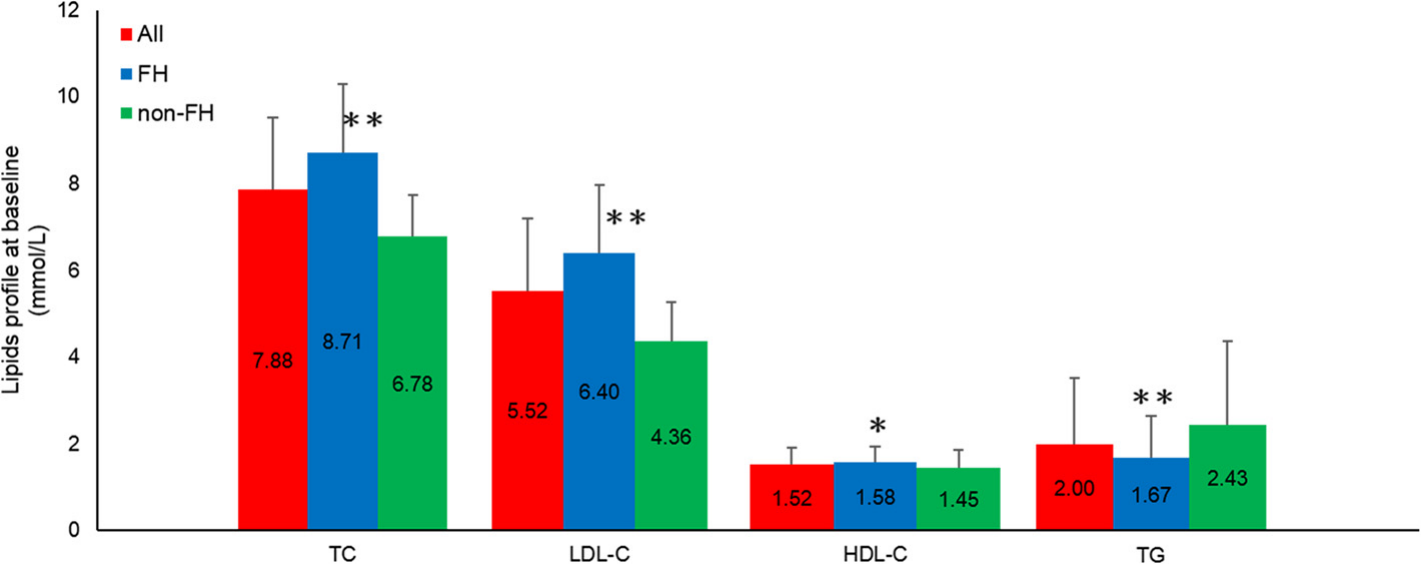
Baseline lipid profiles on no lipid-lowering treatment in study subjects. Data are given as mean and error bars represent SD; *P < 0.005; ** *P* < 0.001 for FH vs. non-FH

Both treatments were well tolerated in 247 patients with good compliance. The baseline characteristics of the study participants are shown in Table 1. There was no significant difference in baseline characteristics between subjects with different statin treatment orders (data not shown). The mean (±SD) age of the study subjects was 55.7 ± 11.1 years and 40.5 % (*n* = 100) of the study subjects were male. There were 140 patients having FH who had significantly higher baseline levels of LDL-C and HDL-C but lower triglycerides than those of non-FH patients (Fig. 1).

**Table 1:** Baseline characteristics of the study participants

	All	Males	Females	*P*
	(*n* = 247)	(*n* = 100)	(*n* = 147)	
Age, years	55.7 ± 11.1	55.0 ± 11.0	56.1 ± 11.3	0.425
FH, *n* (%)	140 (56.7)	53 (53.0)	87 (59.2)	0.336
Hypertension, *n* (%)	118 (47.8)	52 (52.0)	66 (44.9)	0.273
Type 2 diabetes, *n* (%)	48 (19.4)	24 (24.0)	24 (16.3)	0.135
History of CVD, *n* (%)	24 (9.7)	10 (10.0)	14 (9.5)	0.901
Current smoker, *n* (%)	26 (10.5)	24 (24.0)	2 (1.4)	<0.001

*CVD*, cardiovascular disease; *FH*, familial hypercholesterolaemia

**Fig. 2:**
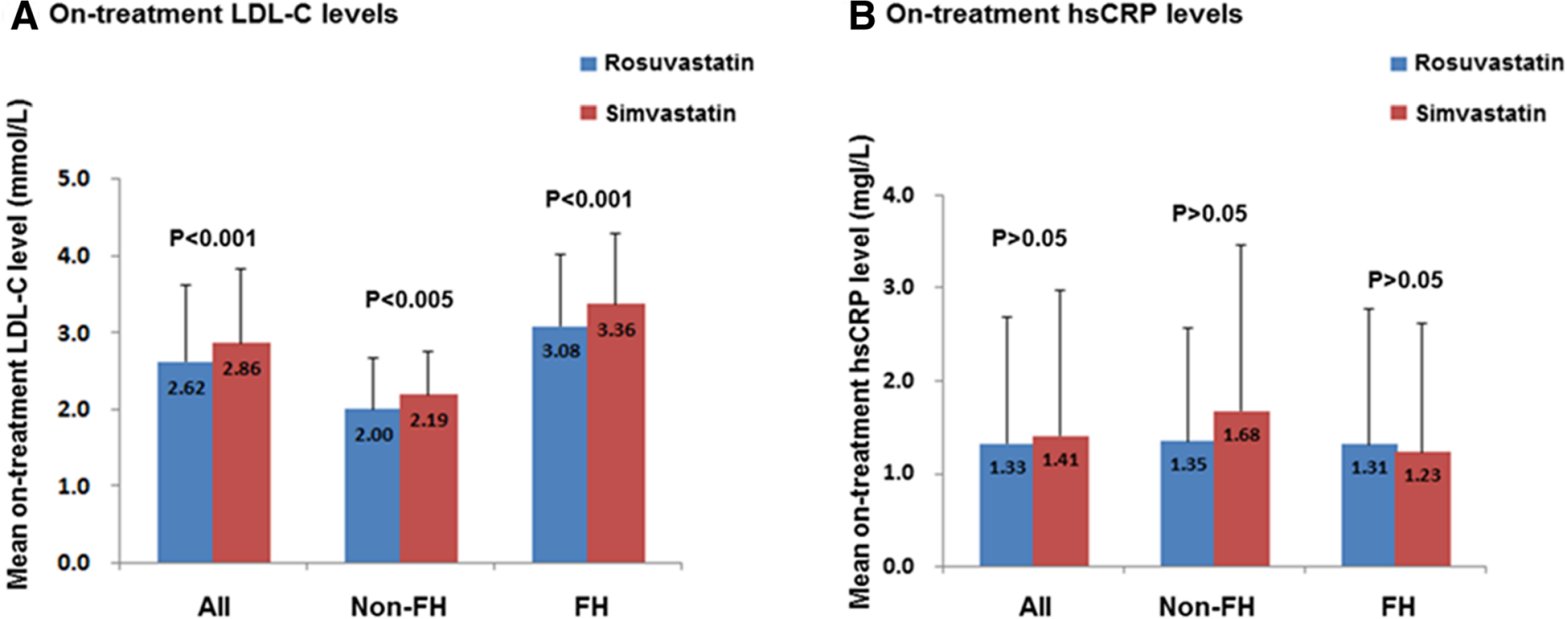
Baseline lipid profiles on no lipid-lowering treatment in study subjects. Data are given as mean and error bars represent SD. (**a**) On-treatment LDL-C leves in all patients and in subgroups of FH and non-FH patients. (**b**) On-treatment hsCRP leves in all patients and in subgroups of FH and non-FH patients

There were significant differences (*P* < 0.001) for rosuvastatin versus simvastatin for the on-treatment LDL-C (2.62 ± 0.99 mmol/L vs. 2.86 ± 0.97 mmol/L,) and the percentage reduction in LDL-C (−52.4 ± 11.9 % vs. -47.7 ± 10.8 %), but not for the on-treatment hsCRP levels (1.33 ± 1.37 mg/L vs. 1.41 ± 1.57 mg/L, *P* > 0.05) in overall subjects and in subgroups of FH and non-FH patients, respectively (Fig. 2). Body weight and waist circumference were not different whilst on the two statin treatments (body weight: 64.1 ± 13.1 kg with rosuvastatin and 64. ± 13.1 kg with simvastatin, *P* > 0.05; waist circumference: 85.9 ± 11.6 cm vs. 85.3 ± 12.6 cm, *P* > 0.05)._

The on-treatment LDL-C and the on-treatment hsCRP levels on rosuvastatin treatment were significantly correlated with the respective values on simvastatin (*r* = 0.812 and *r* = 0.683, *P* < 0.001 for both), whereas there was no correlation between the on-treatment LDL-C level or the percentage reduction in LDL-C in response to either statin and the on-treatment hsCRP levels (*P* > 0.05). There was no difference in on-treatment LDL-C and hsCRP levels with the two statins in patients receiving the statin treatments in a different order of (data not shown).

**Fig. 3:**
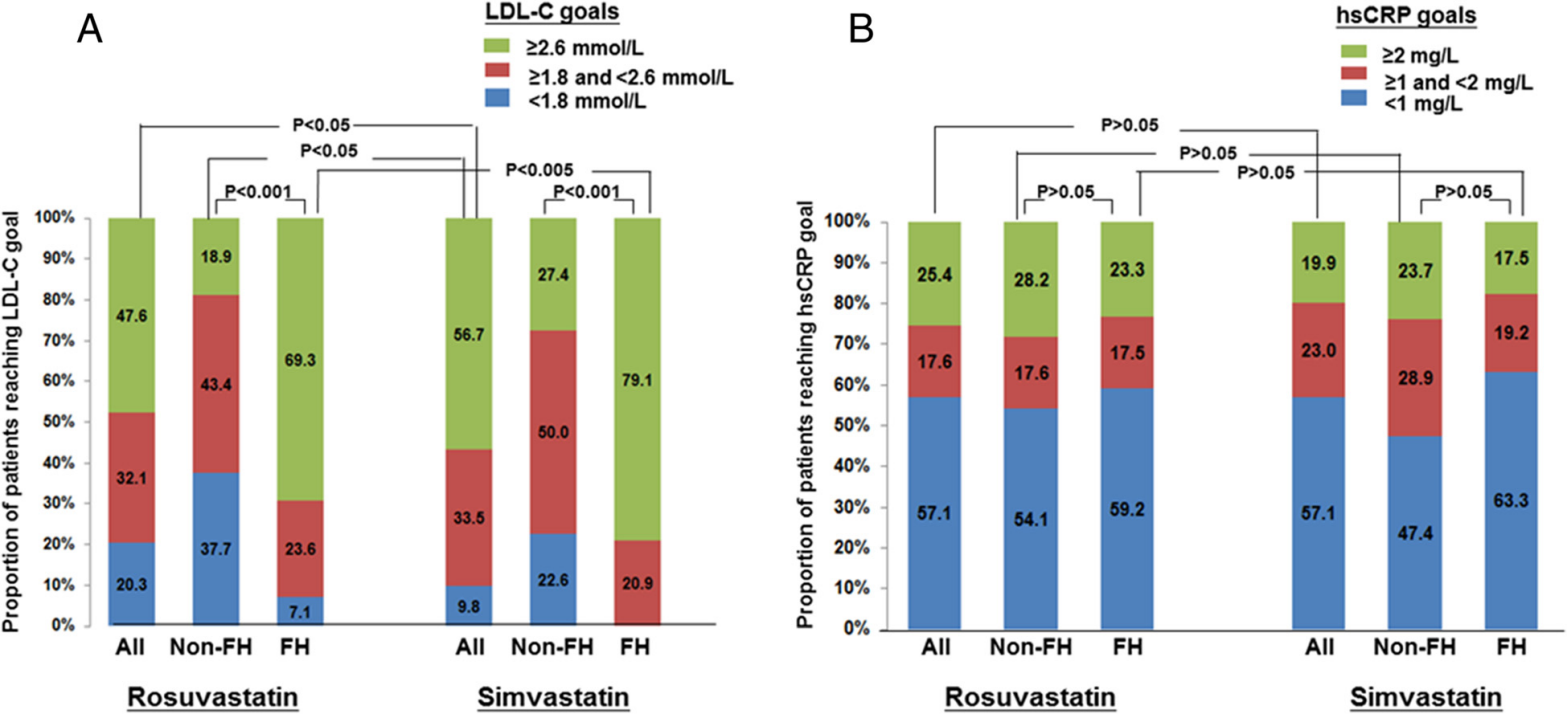
Proportion of subjects with on-treatment LDL-C of <2.6 and <1.8 mmol/L (**a**) and hsCRP <2 and <1 mg/L (**b**) with the two statins

The LDL-C targets of <2.6 mmol/L and <1.8 mmol/L were achieved by 52.4 % and 20.3 % with rosuvastatin compared with 43.3 % and 9.8 % with simvastatin in all subjects (*P* < 0.05). The difference in LDL-C goal attainment with the two statins was more significant in FH patients (Fig. 3). There were higher proportions of non-FH patients achieving the LDL-C goals with both statins than FH patients, as FH patients had higher baseline LDL-C levels (Fig. 3). The proportions of patients attaining hsCRP goals of <2 and <1 mg/L with the two statins or in patients with or without FH were not significantly different (*P* > 0.05) (Fig. 3).

There were 39 % of patients achieving both targets for LDL-C of <2.6 mmol/L and hsCRP <2 mg/L with rosuvastatin treatment compared to 28.1 % patients attaining both targets with simvastatin treatment (*P* < 0.05).

## Discussion

With the advantage of comparing two treatments in the same subjects in a crossover design, this study showed that a significantly (*P* < 0.05) greater proportion of patients achieved LDL-C targets with rosuvastatin 10 mg compared with simvastatin 40 mg in this group of Chinese patients with increased cardiovascular risk. There were 81.1 % and 72.6 % of non-FH patients reaching the LDL-C level of <2.6 mmol/L with rosuvastatin and simvastatin, respectively. However, in patients with FH, lipid-lowering monotherapy with rosuvastatin and simvastatin only enabled 30.7 % and 20.9 % of patients to achieve the LDL-C level of <2.6 mmol/L. Furthermore, there were no patients who achieved the LDL-C goal of <1.8 mmol/L with simvastatin treatment compared with 7.1 % who achieved it with rosuvastatin. These results suggest that higher statin doses or combination therapy is needed to reach LDL-C goals in Chinese patients with FH, as in other ethnic groups [20].

In a parallel group study in Greek patients with hyperlipidaemia, rosuvastatin 10 mg and simvastatin 40 mg had similar effects on the reductions in both LDL-C and hs-CRP and the combination of simvastatin 10 mg with ezetimibe 10 mg also produced similar responses [21]. That finding may suggest that there is less difference in the effects of these doses of the two statins on LDL-C in Caucasians than in Chinese, which would be compatible with the known ethnic differences in rosuvastatin pharmacokinetics [22]. However, recent data from the VOYAGER meta-analysis suggested that 10 mg rosuvastatin is equivalent to approximately 70 mg simvastatin for the effect on LDL-C [23].

Our results showed that there were 57.1 % of patients having an on-treatment hsCRP level of <1 mg/L with both of the statins and only 25.4 % and 19.9 % of patients had hsCRP levels ≥2 mg/L with rosuvastatin and simvastatin, respectively (*P* > 0.05). The hsCRP goal attainment was comparable between patients with or without FH and is greater than in patients in western countries [24], which is probably due to a lower baseline hsCRP levels in East Asian populations such as Japanese and Chinese [25]. Baseline hsCRP levels are largely determined by genetic factors and obesity and both may contribute to the ethnic differences in baseline hsCRP levels [26]. In a population-based prospective cohort study in a general Japanese population, higher hsCRP levels were significantly associated with a higher incidence of future CHD events even when the hsCRP level was below 1 mg/L [27]. Adiposity, especially abdominal obesity, is the strongest predictor of hsCRP concentrations across different populations [25], probably resulting from obesity-induced up-regulation of the cytokines IL-6 and TNF-α which contributes to low-grade inflammatory and hsCRP elevation [28]. As Asians have lower cutoff-points for waist circumference and body mass index to define obesity and metabolic syndrome, as recommended by the World Health Organisation [29], it may also be appropriate to have lower hsCRP goals in these East Asian populations.

Current evidence suggests that there is no relationship between the reductions in LDL-C and in hsCRP in response to the statin treatments in individual patients [14, 30, 31]. Similar results were found in Japanese patients treated with pitavastatin [32], and also with atorvastatin [33]. However, a previous meta-analysis which did not include the JUPITER trial demonstrated a strong relationship between average changes in LDL-C and CRP (*r* = 0.80, *P* <0.001) in healthy subjects and subjects with stable coronary artery disease who were treated with LDL-lowering interventions in placebo-controlled trials [34], but this meta-analysis was performed using mean levels of LDL-C and CRP changes in each trial and this may not reflect the true correlation between the changes in LDL-C and CRP in individuals. Another study from Greece demonstrated correlations between CRP reduction and the lipid-lowering effects of simvastatin within Caucasian patients who were receiving chronic haemodialysis [35], but they may represent an unusual group. The present study did not examine the correlation between the changes in LDL-C and hsCRP in response to the two statins as the baseline hsCRP level was not available which is one of the main limitations of the study. However, the stronger correlation between the LDL-C values on the two statin treatments compared to that between the hsCRP values on the two statin treatments and the wide range of hsCRP values on statin treatments suggests that the hsCRP changes with statin treatments are more variable than the LDL-C changes.

In contrast to achieving maximum lipid-lowering effect after at least 4 weeks treatment, statins appear to exert anti-inflammatory and beneficial endothelial effects more rapidly, even after a single dose [36]. For instance, a single 80 mg loading dose of atorvastatin within 24 h of percutaneous coronary intervention reduced the incidence of peri-procedural myocardial infarction [37]. Our previous data showed that the *ABCG2* 421C > A polymorphism plays an important role in determining the on-treatment level and LDL-C response to rosuvastatin in Chinese patients but this polymorphism had no effect on the hsCRP on-treatment levels in this group of subjects [26, 38]. The recent genome-wide analysis in over 3000 subjects of European ancestry who had been randomly allocated to rosuvastatin 20 mg or placebo daily in the JUPITER study also confirmed that a polymorphism (rs1481012) in *ABCG2* which is in nearly complete linkage disequilibrium (LD) with the *ABCG2* 421C > A polymorphism was the only pharmacokinetic-related variant significantly associated on a genome-wide basis with the LDL-C reduction in response to rosuvastatin [39]. This genetic polymorphism together with several other polymorphisms associated with the LDL-C response to rosuvastatin identified in the JUPITER study had no impact on the rosuvastatin-induced hsCRP reduction after correction for multiple testing [40]. This result further supports the hypothesis that statin-mediated effects on inflammation as measured by hsCRP are independent of statin-mediated effects on LDL-C and less influenced by the statin pharmacokinetics.

Some clinical studies showed that higher doses of statins may reduce the hsCRP levels more than lower doses of statins in patients with diabetes or stable coronary disease [41, 42]. However, our study and the JUPITER study showed that patients with the *ABCG2* 421A variant allele with increased systemic exposure to rosuvastatin had similar hsCRP on-treatment level or hsCRP reduction in response to rosuvastatin as those patients without the variant allele suggesting that some other genetic and/or environmental factors may affect the hsCRP lowering effect in response to statins. The dosage of statins used in our study might be considered as relatively high in some Asian countries. However, previous studies in Japan have shown that rosuvastatin 10 mg results in reductions in LDL-C of approximately 50 % which is similar to our study [43, 44]. In the STELLAR Trial, which compared different doses of rosuvastatin and other statins, the LDL-C reduction with rosuvastatin 10 mg was 45.8 % and with simvastatin 40 mg was 38.8 % in a mainly Caucasian Group [45]. These values are both less than the LDL-C reductions seen in the Hong Kong patients, probably because the STELLAR Trial used an intention to treat analysis whereas the Hong Kong patients were selected for having good compliance with therapy. Furthermore, it was shown that this degree of LDL-C reduction is needed to achieve regression of coronary atheroma in Japanese patients [17].

The present study showed there was no difference in hsCRP levels with the two statins with different potency in reducing LDL-C levels in this patient group. Rosen et al. have reported that although switching to ezetimibe/simvastatin (10/20 mg) was more effective at reducing LDL-C vs. doubling the baseline statin dose to simvastatin 40 mg or atorvastatin 20 mg or switching to rosuvastatin 10 mg, the combination therapy was not superior to statin monotherapy in reducing hsCRP [46]. Interestingly, another study demonstrated that despite having similar lipid-lowering potency, atorvastatin 10 mg resulted in greater reductions hsCRP and its variability compared with simvastatin 40 mg in type 2 diabetic patients [47]. It has been shown that treatment with high-dose (80 mg daily) atorvastatin for 2 weeks had no effect on hsCRP levels in normolipidaemic subjects with normal hsCRP levels although the LDL-C levels were significantly reduced indicating baseline hsCRP levels may play an important role in determining the statin-mediated hsCRP reduction [48].

## Conclusions

In conclusion, this study showed that a significantly greater proportion of patients achieved LDL-C targets with rosuvastatin 10 mg compared to simvastatin 40 mg in Chinese patients with or without FH, but there was no difference in hsCRP levels with the two statin treatments with the majority of patients reaching hsCRP levels of <2 mg/L. The study also suggested that more intensive lipid-lowering treatments are needed to enable more FH patients to reach their LDL-C goals.
